# Advances in engineered models of peri-gastrulation

**DOI:** 10.1016/j.isci.2025.112659

**Published:** 2025-05-14

**Authors:** Diandian Cheng, Christopher T. Clark, Quinton Smith

**Affiliations:** 1 Department of Chemical and Biomolecular Engineering, University of California, Irvine, Irvine, CA 92697, USA; 2 Sue and Bill Gross Stem Cell Research Center, University of California, Irvine, Irvine, CA 92697, USA; 3 Department of Biomedical Engineering, University of California, Irvine, Irvine, CA 92697, USA; 4 Department of Materials Science and Engineering, University of California, Irvine, Irvine, CA 92697, USA

**Keywords:** Bioengineering, Developmental biology, Embryology, Biology of human development

## Abstract

Studying human development presents significant ethical and technical challenges. Yet, the integration of stem cell technology and engineering tools has provided unprecedented insights into early lineage specification and the morphogenetic events that shape human development. Pre-gastrulation models, such as blastoids, replicate aspects of blastocyst formation and implantation, enabling research on early lineage specification and embryo-maternal interactions. Gastrulation models, including 2D micropatterned systems and 3D gastruloid constructs, provide valuable insights into cell differentiation, signaling pathways, and tissue organization during germ layer formation. Beyond gastrulation, post-gastrulation models, including somitoids, mimicking early somitogenesis and axial elongation, offer opportunities for studying segmentation and neural tube formation. Together, these systems enable investigation into the peri-gastrulation stage of mammalian development. Here, we discuss the integration of engineering technologies, including micropatterned substrates, microfluidic systems, and synthetic biology tools, in enhancing the precision of these models, allowing for a greater understanding of the early stages of human development.

## Graphical abstract

**Figure:**
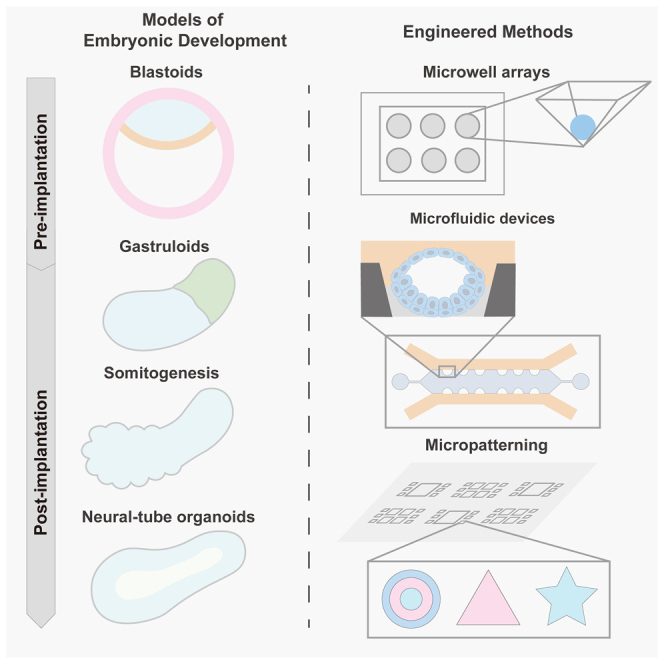

## Introduction

**Figure 1:**
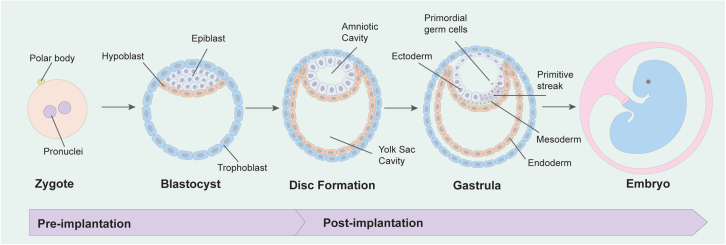
*In vivo* human development Pre-implantation - Post-fertilization, pronuclei containing the genetic material from the fused gametes for single nuclei, giving rise to the zygote. Post-fusion, a series of division events occur, leading to the formation of the morula (a term derived from the Latin word “morus”, which means mulberry). Next, the embryo continues to mature in the peri-implantation stage*,* where the inner cell mass undergoes further maturation upon implantation, forming the hypoblast and epiblast. The epiblast, which is organized at the opposite pole of the hypoblast, will continue to differentiate, forming the amniotic cavity, while the hypoblast will go on to form the yolk sac. In the post-implantation stage, gastrulation proceeds, and progenitors in the epiblast travel through the primitive streak by epithelial-to-mesenchymal transition (EMT), giving rise to mesoderm and endoderm progenitors, resulting in a tri-laminar disk structure (ectoderm, endoderm, and mesoderm) serving as the blueprint for downstream formation of rudimentary organ structures.

At the outset of mammalian development, cells undergo gastrulation, transitioning from a pluripotent stage capable of forming all adult tissues into three germ layers with distinct cellular fates. This event occurs inside a cellular construct known as the blastocyst, a fluid-filled cavity (blastocoel) encased by a layer of trophoblast stem cells (TSCs) and a compacted inner cell mass (ICM) of pluripotent human embryonic stem cells (hESCs) arising from the unspecified morula.^1^ Once formed, the blastocyst implants into the uterine wall, where the ICM forms the fetus (Figure 1). TSCs mature into extraembryonic tissues, including the placenta, umbilical cord, and membranes that protect and support waste and nutrient exchange.^2^ The expanding ICM undergoes amniogenesis, creating a lumenized cavity with an outer amniotic ectoderm and inner epiblast poles.^3^ Following amniogenesis, the human epiblast morphs into a trilaminar disk containing the ectoderm, mesoderm, and endoderm germ layers, which template the vertebrate body plan in a process called gastrulation. The orchestration of pre- and post-implantation morphogenesis remains underexplored in humans due to limited tissue accessibility and ethical oversight. However, hESCs and reprogrammed somatic adult cells to human induced pluripotent stem cells (hiPSCs) enable *in vitro* models of human embryonic morphogenesis and maturation.^4^^,^^5^

Despite advancements in stem cell technologies, existing biomimetic culture platforms lack the structural complexity and compartmentalization inherent in embryonic development. Further integration with microenvironmental and cellular engineering is needed to advance *in vitro* models of embryonic morphogenesis. While native developmental environments involve complex cross-cellular signaling events, tissue-specific differentiation studies in both 2D and 3D contexts provide clearer insights into the signaling events driving development.^6^^,^^7^ These approaches have shed light on fundamental developmental processes and offer valuable insights into the molecular mechanisms underlying human embryogenesis. However, many questions remain unanswered. For instance, can we achieve a more representative organization in 2D and 3D culture systems by controlled mechanical stimulation and the presentation of soluble morphogens? How might cellular signaling gradients influence various combined organizations of multiple cell types differentiating together? How does the presence or absence of specific transcription factors influence the ability of the germ layer and later tissue types to develop and organize themselves? Thus, as we seek to understand the biology of human embryonic morphogenesis, it is essential to continue developing and utilizing engineered systems for *in vitro* environments to mimic the developmental niche. Recent improvements in studying embryonic models have significantly expanded our understanding of early embryo development. These models can mimic many stages of human development, such as the formation of the blastocyst (blastoids), the process of gastrulation (gastruloids), and the formation of more developmentally advanced structures, including somites and the neural tube. Here, a comprehensive overview of recent advances in engineered models of developmental morphogenesis will be explored.

## Blastoids

**Figure 2:**
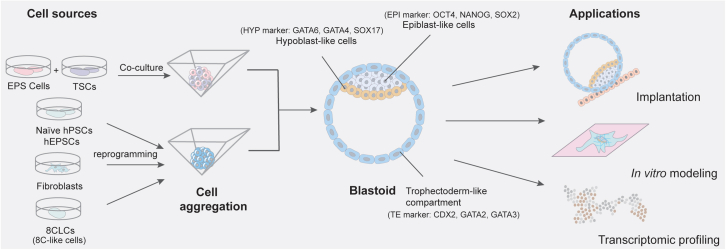
Blastoid formation and extended studies Blastoids formation - Blastoids can be formed through a co-culture of extended pluripotent stem (EPS) cells with trophoblast stem cells (TSCs) or through the aggregation and differentiation of naive pluripotent stem cells (PSCs), expanded pluripotent stem cells (EPSCs), directly reprogrammed stromal cells, or cells mimicking the 8-cell stage of embryonic development. These blastoids recapitulate many hallmarks of *in vivo* blastocysts, including the epiblast (OCT4^+^, NANOG^+^, SOX2^+^), the hypoblast (GATA6^+^, GATA4^+^, SOX17^+^), and the trophectoderm (CDX2^+^, GATA2^+^, GATA3^+^), in addition to an open luminal space. Applications - These models have been used for a variety of applications such as studying implantation, cell signaling pathways, and transcriptomic profiling, to validate their functionality as a benchmark for maternal-fetal interactions.^11^^,^^26^^,^^27^

At one of the earliest stages of development, cells form the blastocyst, a cavitated and polarized structure derived from the morula. The blastocyst contains an outer trophoblast layer and ICM, which will continue to delineate into hypoblast and epiblast layers prior to later cellular specialization. This developmental point is exceedingly hard to study *in vivo* due to ethical concerns as well as the very early and transient nature of this cellular organization, with the blastocyst existing in human development between 5 and 7 days post fertilization, while mice undergo this event at 3.5 days post fertilization.^8^^,^^9^ To aid with investigations into this developmental milestone, recent advances have been made in the creation of blastoids as a model system *in vitro* (Figure 2). These studies leverage stem cells in a naive state, mimicking an earlier developmental stage than cultured hESCs derived from the ICM or hiPSCs. By combining naive stem cells with forced aggregation techniques, tissue constructs that mimic the morphological, transcriptional, and epigenetic markers of natural human blastocysts with over 80% efficiency from both hiPSC and hESC populations have been generated.^10^ Microfabricated pyramidal or U-shaped non-adherent microwells facilitate the controlled aggregation of pluripotent stem cells (PSCs) and have been used to model ICM compaction and subsequent developmental events. Forced aggregation by these technologies enables the control of spheroid size, tissue uniformity, diffusible morphogens, and aggregate cellular mechanics, which in turn dictate differentiation trajectory and morphogenic behavior (Box 1A).^11^^,^^12^^,^^13^^,^^14^^,^^15^^,^^16^^,^^17^^,^^18^^,^^19^ In addition to naive stem cells generated from reprogramming iPSCs and ESCs,^11^ blastoid models have also been produced through direct reprogramming of stromal cells like fibroblasts,^20^^,^^21^ expanded pluripotent stem cells (EPSCs) converted from PSCs,^22^ and utilization of 8 cell-like cells (8CLCs)^23^ and a co-culture of extended pluripotent stem (EPS) cells with trophoblast stem cells (TSCs) (Figure 2).^24^^,^^25^ Lab-made blastoids demonstrated the formation of a blastocoel cavity in concert with a pluripotent epiblast (OCT4^+^), trophectoderm (GATA3^+^), and primitive endoderm (SOX17^+^) cell populations, as measured with both immunofluorescence staining and single-cell RNA sequencing, within seven days of initial cell seeding in aggregation conditions (Figure 2). These cellular constructs have been cultured for up to 21 days, expressing an outer layer of trophoblast (KRT7^+^) cells, an inner layer of epiblast cells (OCT4^+^), and a mesendoderm population (GATA6^+^), similar to Carnegie stage 7 embryos, as evidenced by RNA-sequencing.^26^ Expanding on these and similar findings, Zhao et al. 2025 compared three sequencing libraries of published naive stage cells formed into pre-implantation blastoids and found that the model constructs had clear overlap with human pre-implantation stage cells for epiblast, hypoblast, and trophectoderm-like cells, with the notable addition of post-implantation cell clusters not seen in native pre-implantation stage cell samples (Figure 2).^27^

Box 1 **Engineering approaches to control developmental morphogenesis**

**A. Forced Aggregation -** The generation of blastoids and gastruloid embryonic models relies on the precise control of cell aggregation to mimic early developmental organization. Subsequent to expanding stem cell populations in 2D conditions, there are several platforms that have been leveraged to encourage cell-to-cell interactions in the form of aggregates. U-bottom wells are scalable platforms that offer control over aggregate uniformity with inverted dome-shaped well bottoms that encourage cell aggregation at the well’s center. AggreWell plates, contain microwells that standardize the size and shape of aggregates by confining cells within defined areas similar to a U-bottom but consisting of altered geometry in the aggregation area. This method ensures high-throughput and uniform spheroid formation, which is critical for reproducibility in generating embryonic organoid models. These aggregates can be transferred to different culture environments (rotary,^12^ static,^28^ ECM hydrogel embedded^29^) to further develop into self-organized structures.
**B. Micropatterning -** Micropatterning techniques use photolithography, soft-lithography, or microcontact printing to create defined adhesive regions on culture substrates, controlling cell geometry and spatial organization. Confining pluripotent populations on patterned surfaces enables the study of how colony size, shape, and boundary conditions influence symmetry breaking, axis formation, and germ layer specification. For example, micropatterned stem cells exposed to BMP4 gradients exhibit radially organized germ-layer patterning mirroring gastrulation or are able to undergo morphogenesis mimicking neural tube folding and lumenogenesis.^30^
**C. Microfluidics -** These systems enable precise control over the cellular microenvironment by manipulating fluid flow, chemical gradients, tissue compartmentalization, and mechanical forces. These tools are fabricated using soft-lithography techniques to produce channels, shapes, or valves that allow dynamic culture environments. In the context of studying embryogenesis, microfluidic platforms have been used to generate stable morphogen gradients to deliver localized signaling cues, enabling spatially controlled differentiation and morphogenesis, such as in modeling amnion formation.^31^
**D. Synthetic Biology -** Cellular behavior can be programmed by engineering gene circuits and signal transduction pathways. Through the introduction of synthetic signaling centers, inducible transcription factors, and cell-cell interactions, cell fate decisions and organizational processes can be user-defined. Synthetic circuits have been designed to modulate WNT, NODAL, or BMP pathways, which are necessary for gastrulation processes,^32^ enabling tunable tissue formation and patterning processes. The integration of synthetic biology with established stem cell-derived embryonic models offers additional insight into how signaling factors are propagated and can lead to the creation of synthetic tissues.

**Figure:**
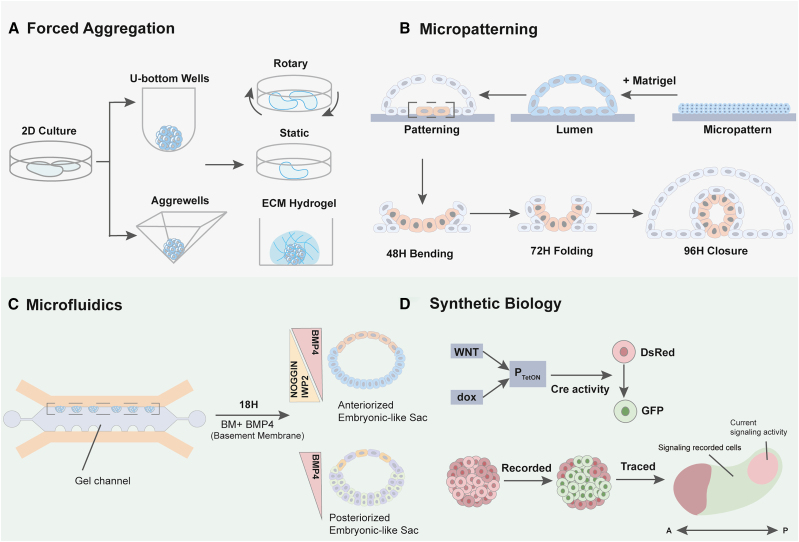

In some studies, blastoids generated from naive stem cell populations have been used to recapitulate implantation stages of development by attaching to stimulated 2D open-face endometrial cells and even recapitulating impaired attachment under contraceptive levonorgestrel addition, demonstrating the functional similarities to native human blastocysts.^11^ With the co-culture of 3D apical-out endometrial organoids and blastoids, a feto-maternal assembloid can be formed. This system was able to not only mimic ICM polarization but also the embryo’s correct organization relative to the endometrium, with the ICM forming at the opposite pole to the site of implantation. In addition, through assembloid culture, endometrial stromal cell fusion was observed with the implanting blastoids.^33^

Although stem cell technologies have allowed for new experimental opportunities, key questions are yet to be answered in this context. For example, work from Zhao et al. (2025) has demonstrated that lab-grown blastoids have significant variation in composition between published sequencing datasets and with notable differences to human embryos.^27^ With the continued application of these systems, blastoid studies could be expanded to study developmental diseases influencing implantation, as well as the role of endometriosis and uterine fibrosis in fertility. Following body plan determination, the developing embryo continues integrating biophysical and chemical signals with gene regulatory networks to initiate progenitor cell sorting and the formation of multicellular tissues with structural and functional compartmentalization. In the following sections will describe engineering approaches used to study gastrulation.

## Engineered gastrulation models

Gastrulation *in vivo* involves several critical events, including differentiation, EMT, and migration, and these processes have been modeled in both 2D and 3D constructs. Due to the complexity of regulating multiple signals in space and time, our understanding of signaling dynamics within mammalian embryos is unclear. Our current insights into signaling cascades that take place during gastrulation have previously come from numerous studies in mouse embryos due to ethical considerations. At the onset of gastrulation, a complex signaling hub is established in the proximal posterior epiblast (Figure 1). BMP4 and NODAL signals are initiated in the extraembryonic ectoderm (ExE). BMP4 signaling then induces WNT signal in both the epiblast and the overlaying visceral endoderm. WNT can stimulate NODAL signaling, where NODAL activates its expression in a feedback loop to help maintain BMP signaling. Meanwhile, antagonists of each signal (CER1 for BMP, LEFTY1 for NODAL, and DKK1 for WNT), which are secreted from the anterior visceral endoderm (AVE), help confine their expression to the posterior region of the embryo where they induce the formation of the primitive streak.^34^ Engineered approaches for studying gastrulation models include creating 3D cell aggregates (Box 1A). Alternatively, gastrulation can be modeled using micropatterns where ECM is administered in a spatially controlled manner (Box 1B). Gastruloids, as *in vitro* models, offer unique insights into those developmental processes, characterized by features such as symmetry breaking, cell polarization, and multi-lineage emergence.^35^^,^^36^

### 2D micropatterned gastrulation models

**Figure 3:**
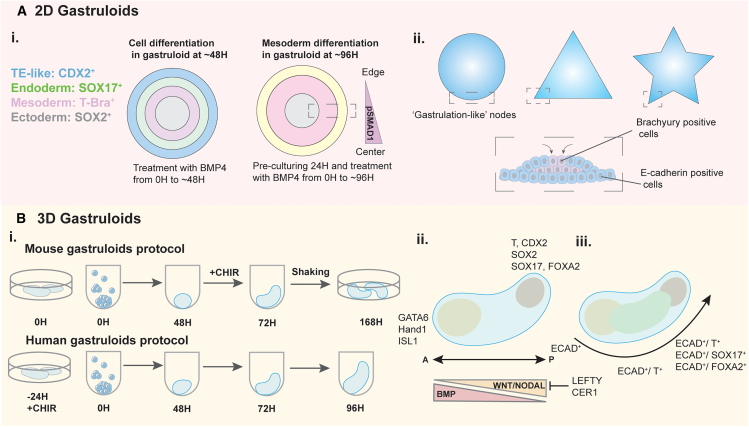
*In vitro* gastrulation models (A) 2D Gastruloids - (i) Unguided differentiation of 2D micropatterned ESCs and iPSCs leads to concentric rings of ectoderm (SOX2^+^), mesoderm (T^+^), endoderm (SOX17^+^), and trophectoderm-like (CDX2^+^) cells radiating outwards arising from a gradient of BMP4 signaling over 48 h of cell culture.^40^^,^^42^ (ii) Cellular tension can be generated through micropattern confinement with higher tension at colony edges and at higher angle vertices (Top).^43^ In some micropattern models, a core of Brachyury positive (T^+^) core can form surrounded by an E-cadherin positive layer of cells (Bottom).^44^ (B) 3D Gastruloids - (i) Protocols of both mouse and human 3D gastruloid formation.^12^^,^^45^ (ii) Schematic of cell signaling gradients in a human gastrulation model.^45^ (iii) Cell-transition processes in mouse gastruloids.^46^ With the treatment of CHIR, ECAD^+^ cells transition to T^+^ cells. Cells expressing ECAD are surrounded by T^+^ cells before both are transported to the end of aggregates, where they cause the formation of an endoderm-like region.

The formation of gastruloids *in vitro* involves the activation of three crucial cell signaling pathways: Wingless Int-1 (WNT), bone morphogenic protein (BMP), and NODAL. These pathways serve as foundational elements for organizing and patterning in 2D and 3D. BMP4, acting as an upstream signaling molecule, regulates the downstream signaling of WNT, which then activates the Activin-NODAL pathway leading to the organization in the differentiating tissues guided by chemical gradients.^37^ Due to the complex environmental niche in which gastrulation occurs, many recent advances have utilized methods of 2D cell culture for germ layer differentiation to monitor individual processes in isolation. One of the first examples of a gastrulation model in 2D relied on restricting cell growth areas through micropatterning extracellular matrix (ECM), leading to geometric confinement. This technique allows for hPSC colony size and shape to be tightly controlled, permitting the study of how tissue geometry influences gastrulation and differentiation kinetics.^38^^,^^39^ Upon stimulation with BMP4, hPSCs patterned on 1-mm circular islands organize into a 2D approximation of gastrulation, forming concentric rings, with an ectodermal (SOX2^+^ and NANOG^−^) center followed by Brachyury (T^+^), which acts as the early primitive streak-like and mesoderm marker, endoderm (SOX17^+^), and extraembryonic lineage expanding out from the core within 48 h (H) (Figure 3A i).^40^ The cellular populations, found here in a planar organization, formed cellular populations similar to Carnegie-stage-7 human gastrula.^41^ After 96H in culture the aforementioned germ layers developed into distinct cellular populations expressing markers for the anterior (T^+^, MIXL1^+^, and EOMES^+^) and posterior (T^+^, WNT8A^+^, MIXL1^-^, and EOMES^−^) portions of the primitive streak.^42^

The utility of micropatterned confined growth conditions has been used to investigate many specific processes in human gastrulation events in high levels of detail. One such area of investigation where micropatterns proved to be of great utility was in high-throughput screening on stem cell cultures to investigate line-specific heterogeneity and differentiation rapidly. Stem cell lines are known to have heterogeneous gene expression levels and differentiation biases that can be costly and time-consuming to optimize for various differentiation targets.^47^ In an effort to streamline this process, micropatterned ECM islands, allowing for dozens of confined ESC colonies in a single well, have been used to rapidly screen stem cell lines for a peri-gastrulation (T^+^ and SOX2^-^) or pre-neurulation (T^−^ and SOX2^-^) lineage specification. This was done using a consistent BMP4-based differentiation, identifying which cell lines are predisposed to these separate developmental trajectories.^48^ Micropatterned stem cell culture has also been extensively utilized to investigate how signal propagation and pathway stimulation interact to influence cellular fate interactions and to understand how human tissues self-organize at this developmental stage.

2D models of gastrulation allow for studies of how the interplay between BMP4, NOGGIN, WNT, and NODAL short-range signaling waves generate cellular patterning events in a Turing-like system of activation and inhibition.^49^ In this signaling paradigm, an initial pulse of an activator leads to the production of the activator and a suppressor that will limit further production. Then, through reaction-diffusion kinetics, a gradient of activator concentration can be formed to spatially pattern an area. In micropatterned gastrulation models, a BMP4 pulse allows for continued BMP4 production as an activator along with NOGGIN, which suppresses BMP4 expression profiles in circular micropatterns, which then inhibits BMP4.^50^ The observation of this patterning method has proven difficult to observe in 3D gastrulation culture but has been readily observed in 2D models.^51^ In hESC culture, the spread of apical secreted NOGGIN was far faster than basal secreted BMP4. This resulted in patterned BMP4 concentrations and germ layer specification in line with Turing patterning models.^52^ This patterning of BMP4 reaction-diffusion-limited signaling has been used to describe the formation of mesodermal (T^+^) and endodermal (SOX17^+^) layers in self-organized patterns as discussed previously into ring like formations in 2D gastrulation studies.^53^ Other works have demonstrated that there is no reaction-diffusion-based Turing-like system of signal control. Instead, an initiated wave of WNT/NODAL signaling propagating from the colony periphery determines the eventual cell fate and has been postulated as a mechanism for the gastrulation-like organization in circular 2D micropatterned systems.^54^ While cell-driven organized patterns like those observed in human development have been produced in a 2D cellular construct, defined signaling gradients have also been investigated to control germ layer patterning *in vitro* to further investigate these mechanisms.

### Microfluidic models

In a system where hESCs were exposed to a microfluidic-driven gradient of BMP4, mesoderm-like MIXL1^+^ cells localized near the BMP4 source of the circular pattern, while ectoderm SOX2^+^ cells localized to the sink end, with little to no observable ring-like structures appearing in the circular micropatterns. This finding was reinforced when a counter-gradient of BMP antagonist NOGGIN was introduced on the opposite side of the device, with stronger polarization and near complete endoderm SOX17^+^ cell polarization to the high BMP4 region of the device.^55^ In planar 2D gastrulation model systems, extraembryonic trophoblast-like CDX2+ regions at the colonies' peripheries have also been observed in both human and mouse ESCs (Figure 3A i),^56^ a striking result as trophectoderm specification occurs during the blastocyst stage *in vivo*. In a BMP4 signaling experiment, trophectoderm-like GATA3^+^ cells were the first to be separated from a pluripotent (SOX2^+^ and NANOG^+^) population, identified as soon as 12H after BMP4 treatment. These cellular identities were then followed by ectoderm specification (SOX2^+^, POU5F1^−^, and NANOG^−^) emerging at 24H, with endoderm (SOX17^+^) and mesoderm (T^+^ and MESP1^+^) populations being observed at 44H into differentiation.^41^ While considerably more rapid than *in vivo*, the time course growth displayed here followed observed germ layer formation events in development, demonstrating the utility of such models for human developmental studies.^4^ Microfluidic model systems have also been used to hPSCs under controlled spatial and biochemical conditions. Specifically, epiblast-like and amniotic ectoderm-like cells were generated in a microfluidic system with controlled administration of BMP, driving the formation of a pro-amniotic cavity and a bipolar embryonic sac. Through a combination of single-cell sequencing and immunofluorescence staining, it was determined that this approach could also lead to the development of human primordial germ-like cells, a process that is stipulated to occur before gastrulation, and at the onset of the formation of the embryonic sac (Box 1C).^31^

### Role of mechanics

In addition to the chemical cues needed for gastrulation supplied through soluble factors in solution, the cellular mechanical environment has been proven to dramatically affect cell signaling, maturation, and eventual tissue formation.^57^^,^^58^ While this mechanical impact on cellular differentiation has been observed in many tissue stages (e.g., neurogenic, myogenic, osteogenic, adipogenic), it has also been investigated in gastrulation.^59^^,^^60^^,^^61^ One of the pivotal signaling events controlling both colony expansion and cellular differentiation is the nuclear or cytoplasmic localization of the Yes-associated protein 1 (YAP), which functions as a co-transcriptional activator to link extracellular signaling events such as mechanical tension and cellular polarity to changes in gene expression and cell function.^62^^,^^63^ In micropatterned gastrulation modules, it was observed that a single knockout of YAP ablated the radial organization of the three germ layers, demonstrating a developmental dependency on YAP for germ layer patterning in planar differentiation.^64^ It was also discovered that in YAP knockout iPSCs, while the directed difference between each of the three germ layers was possible, undirected differentiation between a gastrula-like organization was disrupted, with the ectoderm layer being almost fully disrupted.^65^

In a study where cellular hydrogel substrates were selectively stiffened by light patterning with maskless lithography, iPSCs displayed significant improvements under softened culture conditions with definitive endoderm (FOXA2^+^, SOX17^+,^ and hHEX^+^) and early mesoderm (T^+^ and MESP1^+^) markers showing higher levels of expression.^66^ Similarly, through soft substrate culture of hESCs during mesoderm induction, improvements to mesoderm gene expressions could be observed due to increased β-catenin allowing for higher sensitivity to extracellular WNT signaling for differentiation.^67^ This increased pool of cytoplasmic β-catenin has also been demonstrated on compliant substrate cultured iPSCs for mesodermal induction where the increased mesodermal induction was linked to cytoplasmically retained YAP leading to increased degradation of cytoplasmic β-catenin.^68^ For hESC colonies on circular micropatterned culture, it was also observed that cells in the high-density center of a micropattern express transforming growth factor (TGF)-β receptors to the lateral side, while cells on the periphery localized the same receptors to the apical surface. This structure of receptors allowed for a BMP4-activated radial organization of germ layer formation, mimicking human developmental structures.^69^

As mentioned in previous sections, circular micropatterned islands are extensively useful in investigating stem cell differentiation, but by the implementation of defined shapes, varied tension profiles can be produced throughout a colony. The edge geometry of these micropatterned islands can lead to low tension in a circular or square pattern or be designed to provide high cellular tension in a triangular or other high-angle vertices island (Figure 3A ii).^43^ This force can be measured by cells and responded to through higher-density cytoskeletal networks and increased nuclear YAP localization, leading to changes in cell fate and gene transcription as well as cytoplasmic β-catenin pools.^70^^,^^71^ Micropatterning mESCs in hydrogel molds of defined shapes has given insight into germ layer and extraembryonic tissue formation cues based on colony tension around edges of defined curvature. Similar to circular micropatterned differentiations, the core of these patterns displayed an ectoderm identity (SOX2^+^) followed by mesoderm (T^+^), endoderm (SOX17^+^), and then trophectoderm-like (CDX2^+^) populations forming at the periphery. A greater proportion of trophectoderm-like cells was observed in colonies with higher vertex angles, suggesting that decreased cellular tension, modulated by colony geometry, promotes trophectoderm specification.^72^ In iPSC colonies grown on triangular geometries, which elicit regions of increased tissue tension at the vertices over circular or unconfined growth conditions, local mesoderm (T^+^) specification and gastrulation-like morphogenic behavior through tension-regulated Src-mediated β-catenin phosphorylation were found.^44^ The tension-based regulation of this pathway was confirmed using a mutant β-catenin that was unable to recapitulate the mesodermal induction without exogenous WNT3A addition to the culture environment.^73^ These tension-based cues for human mesodermal induction have also been carried down to later-stage cardiovascular differentiation where cells cultured at high-tension edge regions displayed higher levels of endothelial (VECAD^+^) and pericytes (SM22α^+^) like cells, or troponin positive cardiac cells in the center of micropatterns.^74^^,^^75^

The role of tissue and microenvironmental mechanics in modulating developmental pathways is an emerging feature of embryonic development, with the mechanosensitive β-catenin pathway required for gastrulation *in vivo.*^76^ Inhibiting non-muscle myosin II-based contractility prevents tissue invagination, leading to depleted nuclear β-catenin localization and suppressed mesodermal specification during gastrulation. Remarkably, β-catenin localization and gastrulation defects can be rescued by applying a magnetic field ectopically to embryos injected with ultramagnetic liposomes.^77^ This indicates that local tissue strains can initiate developmental signaling and instruct embryonic morphogenic processes. While 2D micropatterned models exhibit radially symmetrical germ layer organization and provide deep insights into how chemical and physical properties influence cellular functionality, they fail to replicate axial organization during the gastrulation process *in vitro*. This limitation arises because gastrulation is inherently a 3D process, requiring dynamic cell movements, symmetry breaking, and coordinated morphogen gradients that cannot be fully captured in a 2D environment. To address these challenges, 3D gastruloid models have been developed, enabling the study of axial elongation, germ layer patterning, and the emergence of spatially restricted signaling centers. In the following section, we discuss how these engineered models have been used to recapitulate key features of embryonic development, providing mechanistic insights into early lineage specification.

## 3D gastruloids

3D gastruloids, formed from aggregates of PSCs, have mimicked symmetry breaking and germ layer specification characteristics of the vertebrate embryos. To date, numerous studies have focused on mESCs where mouse gastruloids are formed through a 24H pulse of WNT agonist CHIR99021 (Chiron/CHIR) (48H–72H) (Figure 3B i).^12^^,^^78^^,^^79^ Aggregates formed from mESCs initiate elongation around 96H, progressing to form anterior-to-posterior (AP) and dorsal-to-ventral (DV) body axes.^12^ In recent years, there have emerged many studies discussing human gastruloids with early models using hESCs (Figure 3B i).^45^ Although both human and mouse models undergo similar developmental steps, there are distinct differences in both structure formation and timelines that can be investigated with 3D gastrulation studies. Notably, human gastruloids begin to break symmetry approximately 48 h after initiation, reaching maximal elongation at around 72–96 h. This developmental pattern closely resembles that of Carnegie stage 9 embryos, demonstrating the formation and organization of three germ layers.^80^ This groundbreaking study serves as a foundational framework for further investigations into human embryonic development; however, there are still some limitations in forming 3D gastruloids *in vitro*. For instance, models exhibit a short timeline of extension, lack neural tissue emergence in the anterior region, and lack extraembryonic structures.^45^^,^^78^^,^^81^ In summary, the investigation into mouse and human gastruloids has led to significant insights into early embryonic development. By expanding upon existing engineered methodologies and exploring novel approaches, researchers are pushing the boundaries of our understanding of gastrulation and embryogenesis.

### Cell signaling in 3D gastruloids

In 2D gastruloids, BMP4 plays a vital role in pattern formation, resulting in a distinct ring-like structure representing the trilaminar disk and extraembryonic cells.^40^ Although BMP4 drives patterned polarization and symmetry breaking in a model of the human epiblast,^82^ it does not exert a significant influence on 3D human gastruloid formation. However, the lack of BMP causes the failure of primitive streak formation and affects the gastrulation process *in vivo.*^34^ In both mouse and human 3D gastruloid models, Brachyury expression is partially co-expressed with CDX2,^83^ marking early posterior symmetry breaking and mesodermal specification, while endoderm markers (SOX17^+^, and FOXA2^+^) localize posteriorly. Conversely, anterior regions in this system exhibited expression of cardiac mesoderm markers (such as GATA6^+^, Hand1^+^, and ISL1^+^) (Figure 3B ii).^12^^,^^45^ Manipulation of the WNT pathway has been shown to induce mesoderm specification in gastrulation-like processes.^84^ Brachyury can cooperate with WNT signaling and subsequently polarize to the posterior area, breaking symmetry in gastruloids.^78^ Treatment with BMP4 drives polarization of Brachyury by upregulating WNT3 and NODAL signaling centers, resulting in embryo-like structures that show AP and DV patterning akin to embryonic day (E) 9.0 mouse embryos.^85^

Although there have been numerous studies demonstrating how vital WNT signaling is during gastrulation, some embryonic patterning systems remain unclear. Currently, the emergence of synthetic biology provides a new direction to understand the mechanisms governing cell patterning. Using a synthetic ‘signal-recording’ gene mechanism, a posterior pole of WNT activity can be traced to understand how WNT responds to axial organization. A gene circuit designed between the WNT signaling pathway and the inclusion of a small molecule, a destabilized doxycycline (dox)-dependent transcription factor (rtTA), together initiates the transformation of a fluorescent signal, aiding in tracing WNT activity (Box 1D). With this tool, it was found that early WNT activity predicts a cell’s location along the AP axis even before polarization occurs.^32^ Similarly, a synthetic organizer was programmed from a fibroblast cell line and established to differentially self-assemble around mESCs dependent on cell adhesion.^86^ These cells were engineered as inducible secretion centers, which allowed the generation of WNT activity gradients. The utilization of synthetic organizer cells can help researchers observe how wide of a dynamic range WNT can have to induce cell lineages along the AP axis.

Notably, BMP4 stimulation directly induces the expression of the WNT3A gene,^87^ facilitating its coordination with WNT signaling to proficiently pattern ectoderm *in vitro.*^88^ Furthermore, Activin has been identified as an additional inducer of NODAL expression, highlighting a feedback loop within the NODAL signaling cascade. The intricate modulation exerted by Activin-NODAL signaling acts as a modifier, finely regulating the WNT pathway to facilitate both mesoderm and endoderm patterning processes.^87^ Optogenetic manipulation has been leveraged to regulate the duration of NODAL signaling during gastrulation, demonstrating how important NODAL is to achieving cell fate specification.^89^ Additionally, the canonical receptors of fibroblast growth factor (FGF), the Ras/Erk and PI3K/Akt signaling cascades were found to have control over axial elongation in mouse gastruloids.^90^ An Akt signaling gradient regulates cell proliferation at the posterior, promoting tissue elongation. Meanwhile, Erk signaling primarily modulates gene expression related to cell-cell adhesion, including Snail and E-cad expression, thereby influencing cellular shape changes during gastrulation. At the same time, Erk signaling also drives the presomitic mesoderm (PSM) and precardiac mesoderm fates during spatial differentiation in gastruloids development. FGF also plays a crucial role in facilitating BMP-induced mesoderm (T^+^) differentiation.^83^ In contrast, the combination of Activin-A and BMP4 promotes mesendoderm differentiation. While the role of the FGF signaling network remains less characterized *in vivo*, FGF is known to drive EMT, a key process in gastrulation.^34^ Overall, synthetic biology technologies open a powerful window into controlling and tracing the development of asymmetric patterns *in vitro*. These insights shed light on how signaling cascades trigger self-organization in 3D gastruloids and provide a platform to study the intricate molecular interplay governing embryonic patterning and morphogenesis *in vivo.*

### Cell-adhesion in 3D peri-gastruloids

In 3D gastruloids, symmetry breaking and cell fate transitions are orchestrated by intricate molecular mechanisms, where cell-cell adhesion properties play a pivotal role.^91^ However, it has been observed that high levels of cell-cell adhesion/contraction inhibit cellular migration during gastruloid formation and fail to elongate. Indeed, exogenous WNT activation with CHIR9901 exposure can disturb E-cadherin expression and promote elongation.^46^ The development of mesoderm specification and promotion of the WNT pathway is triggered through the high cell-adhesion tension upon the release of β-catenin, an intracellular signal transducer in the WNT pathway, dependent on transcriptional activity.^44^ Furthermore, cell-cell junctions have been mediated by E-cadherin to induce mesendoderm (T^+^ and FOXA2^+^) organization and break symmetry through cell migration (Figure 3B iii).^46^ Notably, under the influence of WNT signaling, this process mimics the epithelial organization observed during gastrulation in mammalian development, shedding light on cell state transitions.

The initial cell density correlated with cell-cell adhesion also plays a critical role in both 2D and 3D model formation. As mentioned before, research indicates that in 2D micropatterns, spatial responses to TGF-β Ligands are constrained by cell density, with YAP/TAZ expression showing a lack of nuclear localization as cell density increases.^69^ This underscores the pivotal role of cell-cell communication in driving self-organization within structure formation. For example, lumenogenesis and apicobasal polarization of the epithelium are restricted to PSC aggregates of sufficient size (100 cells/well vs. 25 cells/well),^19^ and apical restriction of E-cadherin can delay lumen opening,^92^ suggesting tension and cell-cell junctional integrity are necessary to initiate these nascent morphogenic processes. In addition to E-cadherin-based cell-cell adhesion, associated signaling of the Rho family of GTPases (RhoA, Rac1, and Cdc42) regulates cytoskeletal assembly. Indeed, hPSC aggregate compaction and epithelialization are impaired when actin polarization or non-muscle myosin II contractility is inhibited.^11^ These *in vitro* systems of early human development highlight the combination of biochemical and mechanical factors needed to drive embryonic morphogenic events and corroborate the role of cytoskeletal dynamics in early development, as reviewed extensively elsewhere.^93^

## Post-gastrulation models

**Figure 4:**
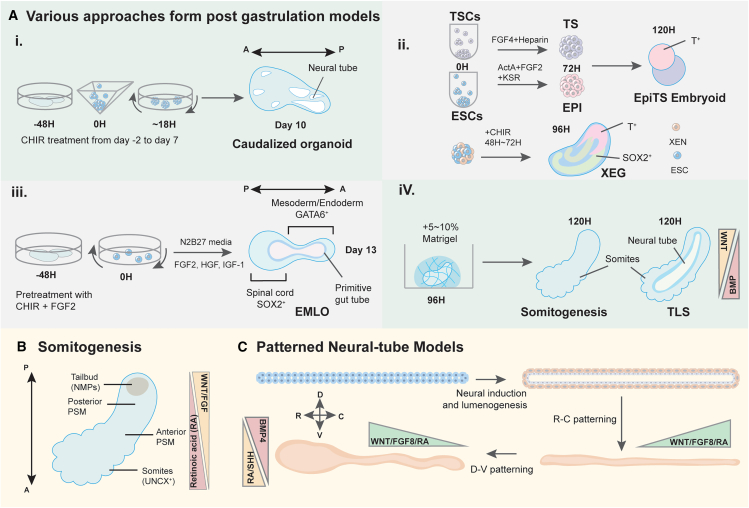
Generation of post-gastrulation models (A) Various approaches to form post-gastrulation models - (i) Human caudalized organoids, being induced by a certain pivotal threshold of WNT agonist (CHIR), were formed with axial elongation and neural tube morphogenesis.^28^ (ii) Trophoblast stem cell (TSC) aggregates (TS) and epiblast-like (EPI) aggregates formed on hydrogel microwells were combined to form a structure termed EpiTS embryoid in order to study axial morphogenesis^19^; XEN-enhanced gastruloids (XEGs) consisted of mESCs and extraembryonic endoderm (XEN) cells exhibit neuroepithelial structures.^94^ (iii) hiPSCs derived elongating multi-lineage organized (EMLO) gastruloids.^95^ (iv) A low percentage of Matrigel embedded with mouse gastruloids can further induce somites and neural-tube formation.^96^^,^^97^ (B) Somitogenesis - Schematic of somitogenesis structure. WNT and FGF signaling form a posterior-to-anterior gradient to regulate PSM differentiation during somite formation, whereas RA activity exhibits an opposing gradient.^100^ (C) Patterned neural-tube models - Formation of microfluidic Rostral (R)-Caudal (C) and Dorsal (D)-Ventral (V) patterned neural tube-like structure.^101^

Post-gastrulation models in developmental biology have opened new avenues for exploring previously inaccessible aspects of early human development and overcoming limitations inherent in existing systems (Figure 4). For example, researchers established an axially elongated caudalized organoid with CHIR treatment using either hiPSCs or hESCs, consistent with the architecture of spinal cord development (Figure 4A i).^28^ Some have strived to induce gastruloids without exogenous WNT activation, aiming to generate anterior neural tissues that were lacking in early models.^19^ Others have explored the formation of gastruloids consisting of mESCs and extraembryonic endoderm (XEN) cells,^94^ or TSCs^19^ to develop brain-like models (Figure 4A ii). Additionally, novel protocols such as the dual-fate neuromuscular organoid (NMO) method have been modified to form elongating multi-lineage organized (EMLO) human gastruloids, offering insights into mesendoderm combined with a trunk-like region that represents neuromuscular tissue (Figure 4A iii).^95^ Furthermore, the utilization of Matrigel, a mouse sarcoma ECM blend, has emerged as a versatile approach for forming structures like somites,^29^^,^^96^^,^^97^^,^^98^ the neural tube,^97^^,^^99^ and trunk-like models (Figure 4A.iV).^97^ These converging methods represent a significant step forward from the initially established gastruloids, providing model systems with more mature embryonic architecture, enhancing our ability to recapitulate and study early embryo development *in vitro*.

### Somitogenesis

During embryonic somitogenesis, which is influenced by high FGF/WNT signaling and low BMP signaling, a gradient of FGF/WNT pathway activity controls cellular transformation along the AP axis. This signaling induces the formation of somites, which are segmented blocks of paraxial mesoderm. These somites have attracted considerable attention because of their crucial role in the development of diverse structures, including vertebrae, skin, rib cage, and skeletal muscle *in vivo.* The segmentation clock is an oscillator that controls the timing of somite formation and travels from the posterior unsegmented region, called the presomitic mesoderm (PSM), to the anterior.^102^ This process demonstrates how waves of signaling gradients can induce patterning along the embryo’s AP axis.

Based on these developmental stages, by placing mouse gastruloids in a 5–10% Matrigel solution at 96H, researchers have been able to mimic the process of somitogenesis *in vitro.*^96^ Following this protocol, human somite models have emerged as a significant resource.^29^^,^^98^^,^^103^ Human PSC aggregates embedded in 10% Matrigel can give rise to epithelial somite-like structures.^103^ Signaling waves from WNT, NOTCH, and FGF travel periodically along the AP axis (∼2H in mouse^96^ and ∼5H in human models^98^^,^^103^) during somite formation showing PSM markers (TBX6^+^ and HES7^+^) surrounded by the appearance of NMPs (SOX2^+^ and T^+^) at the end of the somitoids. PSM regions extended to the anterior region, whereas the somite marker UNCX^+^ was expressed exclusively anteriorly (Figure 4B). In comparison to initial gastrulation models, the developed structures demonstrate rostral-caudal patterning and generate somites along the AP axis (Figure 4C). However, Matrigel, which provides an advantageous mechanical environment, helps the promotion of axial elongation and segmentation but does not contribute to the epithelialization of forming segments.^98^ A model called an axioloid exhibits a better demonstration of FGF, WNT, and retinoic acid (RA) signaling components with the addition of Matrigel. Neither Matrigel nor RA alone can achieve fully epithelialized somites with proper apical-basal polarity, showing that RA may be in synergy with Matrigel to promote somite morphogenesis. Alternatively, some research has formed somitoid models without Matrigel culture.^29^ These models exhibit the development of somite-like epithelial structures from PSM to somites without the inclusion of normal AP patterning. Furthermore, to overcome the limitation of high efficiency and consistency of models, some bioengineering tools emerged to produce somite-like structures using micropatterns.^104^ Cysts consisting of epithelialized PSCs were folded from micropatterned epiblast models on a glass coverslip and induced into single-lumen neural tube models to study the axial patterning of somites. By studying somitogenesis *in vitro*, researchers aim to gain a deeper understanding of embryonic development and potentially uncover insights relevant to regenerative medicine and developmental disorders.

### Neural tube and trunk-like structures

During mammalian brain and spinal cord development, the neural tube initially forms as ribbon-like structures that gradually roll and fuse together, ultimately leading to neural tube closure. This process originates from neuroepithelial cells migrating along the AP axis. Through micropatterned ECM-driven geometric confinement, followed by Matrigel overlay and neural induction, pluripotent cells transition from a flat layer to a luminal-enclosed structure resembling the early neural tube (Box 1C).^30^ This neural model follows human developmental trends with bending, folding, and closure of the differentiating ectodermal cells. These principles have been used to drive somitogenesis and to model mesodermal developments in the neural tube. In one such study, an antiparallel gradient of FGF and WNT against RA was used to form circular somite-like structures on a chip where cells were contained in a PDMS growth channel and encapsulated in a Geltrex overlay. The resulting construct displayed progression in structure formation starting from the rostral (high retinoic acid) to caudal (low retinoic acid) ends of the device in a biomimetic model of neural tube development.^105^ Using these gradients, full patterning of neural tube cells ranging from early forebrain to spinal cord cells was conducted in a single construct (Figure 4C).^101^ In these devices, a microprinted Geltrex pattern of high aspect ratio (long and thin) region permitted a 2D representation of the tube-like developmental area of neural tube formation. Cells differentiated in these conditions showed Rostral-Caudal polarization with patterning of the forebrain (OTX^+^), hindbrain (HOXB1^+^), and spinal cord (HOXB4^+^, HOXC9^+^), developing in a seven-day differentiation scheme. Similarly, if the Geltrex micropatterned regions were instead placed as small circular regions along the morphogen gradient, these cell types developed separately in the same organization, suggesting cell-contact-independent differentiation coordination.^101^ Similarly, in a hESCs device with a gradient of WNT controlling GSK3i concentration provided perpendicular to the long axis of a confined cellular population, clear regions of the hindbrain, midbrain, and forebrain form down the WNT concentration profile.^106^

In 3D culture conditions, a suspension of singularized mESCs embedded in Matrigel was able to differentiate to an epiblast-like stage and then self-organize into neural tube organoids.^99^ These organoids exhibit diverse cell types, including midbrain (OTX2^+^), hindbrain (GBX2^+^, HOXB1^+^), and spinal cord (PAX6^+^, HOXB^+^), spanning the AP axis, mirroring mammalian development *in vivo*. It has been shown that there is a relationship between axial elongation and HOX gene expression in both mouse and human models, but the mechanisms involved need to be further studied.^28^^,^^99^ Intriguingly, the spatial elongation of neural tube organoids isn’t influenced by NMPs in mouse models, whereas the elongation of human organoids correlates with the emergence of NMPs.^28^ With controlled activation of WNT signaling, NMPs can be consistently produced, aiding in the extension of the entire organoid. High levels of WNT signaling have been demonstrated to inhibit neuronal cell types (SOX2^+^ and T^+^) while inducing the caudal region (CDX2^+^), which corresponds to mouse neural tissues formed in the absence of WNT activation.^107^ Additionally, embedding mouse gastruloids in 5% Matrigel at 96H induces trunk-like structures (TLSs), which consist of neural tube and somites.^97^ This provides a platform to study the mechanism of post-implantation embryogenesis and shows the key features of neural tube and somitogenesis. Thus, we can observe how biochemical mechanisms contribute to neural tube formation, guiding our understanding of mammalian embryogenesis for future research directions.

## Conclusion

In this review, we discuss how engineering approaches have been leveraged to develop models of the blastocyte (blastoids), gastrulation (gastruloids), and post-gastrulation structures. Blastocyst models provide a platform to study maternal-feto interactions, while gastruloid models have uncovered mechanisms underlying germ layer formation, cell differentiation, and self-organization. 3D gastruloid models have emerged as a promising avenue for studying embryonic morphogenesis for a more complex and complete view of human development. Characterized by their ability to mimic the spatial organization and developmental processes of early embryos, these models offer unique insights into mechanisms driving symmetry breaking, cell polarization, and multilayered emergence. The orchestration of signaling pathways like WNT, BMP, and NODAL in 3D gastruloids mirrors developmental processes observed *in vivo*, underscoring their potential for studying human embryogenesis. In 2D gastrulation models, extensive studies have revealed the interplay of signaling molecules and cell-cell interactions, elucidating the role of morphogens like BMP4 and WNT in driving germ layer patterning. These models not only recapitulate key events of human gastrulation but also highlight the role of mechanosensitive signaling pathways, including YAP and β-catenin, in regulating cellular differentiation and tissue organization. Newly emerged post-gastrulation models have provided insight into post-implantation developmental processes, such as somitogenesis and neural tube formation, compensating for the limited structures developed in gastruloids. Researchers have achieved remarkable progress in recapitulating complex tissue organization and morphogenetic events *in vitro* by leveraging techniques like micropatterning ECM islands and hydrogel encapsulation. Nonetheless, the advancements discussed in this review highlight the significant progress made in modeling early mammalian embryogenesis *in vitro*.

## Perspective

Post-fertilization, the onset of morphogenesis initiates following cleavage divisions and compaction, leading to the formation of the blastocyst, composed of approximately 100–200 cells. This structure differentiates into supportive extraembryonic tissues, including the placenta, and the ICM, which establishes the body-axis plan. Upon uterine implantation, the embryo undergoes a complex, choreographed developmental process, which has historically been a “black box” in human biology. 10–20% of pregnancies end in miscarriage (with nearly 80% occurring in the first trimester). Blastoid models, which mimic pre-implantation stages, provide a promising avenue to investigate implantation dynamics and early pregnancy loss.^108^ Similarly, gastruloid models offer a window into gastrulation-stage pathologies, facilitating the study of congenital anomalies and genetic disorders. Engineered *in vitro* embryo models hold significant promise for advancing reproductive medicine. These systems can help address infertility, improve the success rates of *in vitro* fertilization (IVF), and serve as platforms for screening teratogens and pharmaceuticals to predict developmental toxicity. They can also be leveraged to study congenital diseases and neural tube defects, such as spina bifida, as well as cardiac defects and syndromes like Klippel-Feil and Holt-Oram syndromes, which are implicated in impaired somite formation and mesoderm patterning.

Recent advancements in stem cell technologies, coupled with engineered platforms have enabled the generation of models mimicking blastocyst and gastrulating tissues. While promising, there are numerous ethical and experimental limitations to these *in vitro* derived peri-gastruloid systems. To date, blastoids cannot mimic earlier embryonic stages and according to mouse experiments, blastoids lack the ability to develop to the fetal stage upon implantation.^18^ Gastruloid models in 2D and 3D have limitations in culture duration, and questions arise on how adding exogenous growth factors mimics *in vivo* processes, which induce self-organization. While Matrigel aids in the formation of these developmental structures, this ECM is complex, with batch-to-batch variability that can lead to inconsistent experimental results. Finally, differences in experimental protocols and efficiency based on stem cell sources continue to contribute to significant variations in experiments and low reproducibility.

However, with the integration of engineering tools, such as micropatterning, user-defined biomaterials, microfluidics, gene editing, and synthetic biology, we can further refine blastoid and gastruloid models. For example, dynamic mechanical cues with user-defined hydrogels can replace Matrigel and be integrated into these platforms to better mimic the changing mechanical context of the early embryo.^109^ Microfluidics tools can be further leveraged to drive embryonic patterning, and even the adoption of machine learning and advanced imaging techniques can allow further understanding and reproducibility of these systems.^110^ Considering these advances, several outstanding questions and challenges remain. To what extent can these models faithfully replicate pre- and post-implantation developmental stages? Can “omics” approaches be integrated to capture real-time developmental dynamics? Moreover, the field lacks standardized criteria defining the minimal molecular, morphological, and functional benchmarks required for these models to be considered reliable surrogates for clinical testing. As these technologies advance, it is imperative to establish clear ethical guidelines governing their use. Responsible adoption requires rigorous ethical oversight, standardization and validation of protocols, and ongoing dialogue among scientists, ethicists, and policymakers.

## Acknowledgments

Q.S. acknowledges support from the 10.13039/100000002National Institutes of Health (R35 grant no. GM151099), the Hanna Gray Fellowship Program from the Howard Hughes Medical Institute (grant no. GT15187), and The Pew Chartable Trust (Biomedical Scholar).

## Author contributions

Conceptulization, D.C., C.T.C., and Q.S.; writing-original draft, D.C., C.T.C., and Q.S.; figure crafting, D.C.; writing-reviewing and editing, D.C., C.T.C, and Q.S.; supervision, Q.S.

## Declaration of interests

The authors declare no competing interests.
